# Influence of Humans on Evolution and Mobilization of Environmental Antibiotic Resistome

**DOI:** 10.3201/eid1907.120871

**Published:** 2013-07

**Authors:** William H. Gaze, Stephen M. Krone, D.G. Joakim Larsson, Xian-Zhi Li, Joseph A. Robinson, Pascal Simonet, Kornelia Smalla, Mohammed Timinouni, Ed Topp, Elizabeth M. Wellington, Gerard D. Wright, Yong-Guan Zhu

**Affiliations:** Exeter University, Truro, UK (W.M. Gaze);; University of Idaho, Moscow, Idaho, USA (S.M. Krone);; University of Gothenburg, Göteborg, Sweden (D.G.J. Larsson);; Health Canada, Ottawa, Ontario, Canada (X.-Z. Li);; Zoetis Animal Health, Kalamazoo, Michigan, USA (J.A. Robinson);; Université de Lyon, Ecully, France (P. Simonet);; Julius Kühn-Institut-Federal Research Centre for Cultivated Plants, Braunschweig, Germany (K. Smalla);; Pasteur Institute of Morocco, Casablanca, Morocco (M. Timinouni);; Agriculture and Agri-Food Canada, London, Ontario, Canada (E. Topp);; University of Warwick, Coventry, UK (E.M. Wellington);; McMaster University, Hamilton, Ontario, Canada (G.D. Wright);; Key Laboratory of Urban Environment and Health, Institute of Urban Environment, Chinese Academy of Sciences, Xiamen, China (Y.-G. Zhu)

**Keywords:** Antimicrobial resistance, environment, horizontal gene transfer, evolution, water, soil, plasmid, sewage, bacteria

## Abstract

The clinical failure of antimicrobial drugs that were previously effective in controlling infectious disease is a tragedy of increasing magnitude that gravely affects human health. This resistance by pathogens is often the endpoint of an evolutionary process that began billions of years ago in non–disease-causing microorganisms. This environmental resistome, its mobilization, and the conditions that facilitate its entry into human pathogens are at the heart of the current public health crisis in antibiotic resistance. Understanding the origins, evolution, and mechanisms of transfer of resistance elements is vital to our ability to adequately address this public health issue.

Antimicrobial drug resistance is caused by microbial gene products that attenuate the activity of an antibiotic in an otherwise drug-sensitive organism. Mechanisms involved include modification of the drugs to less toxic derivatives or avoidance of their action by exclusion, target modification, and mutation of target-associated genes. The biologic effect of these resistance determinants is often highly dependent on genetic and organismal context; thus, resistance genes may be latent under certain conditions, only to be activated in others. Movement of these genes from 1 organism to another or an increase in their expression, for example, can trigger a resistance phenotype (*1*). Therefore, to understand its evolution and potential impact of resistance, a broad view of what constitutes resistance must be taken.

Understanding is increasing that much of antimicrobial drug resistance originates in environmental bacteria that do not cause human disease (*2*,*3*). These organisms have evolved over millennia to sense, interact with, and metabolize small molecules and as a result have developed a plethora of mechanisms to modulate the activities of these compounds. The associated genes often offer some broader selective advantage and consequently have been mobilized and horizontally transferred to other microorganisms sharing their ecologic habitat. This dissemination increases the total effect of resistance determinants within the broad collection of environmental bacteria. Discharges of chemical agents (drugs, disinfectants, heavy metals, and other pollutants) into the environment can accelerate the lateral movement of resistance genes across bacterial populations by increasing selective pressure for maintenance of mobile genetic elements (MGEs) (*4*) or by increasing the rate of gene transfer (*5*). Furthermore, humans have created environments with unprecedented mixing opportunities between environmental bacteria and human pathogens in the presence of such selective agents through, for example, sewage and waste water treatment plants, chemical production factories, and the practice of spreading manure on farmland. These opportunities provide conditions that greatly facilitate gene mobilization.

The result is a perfect storm of opportunity for bacterial human pathogens that exploits millions of years of evolution, uncounted microbial generations, and modern human activity. We outline some key aspects of our current understanding of the environmental antibiotic resistome, its mobilization, and the conditions that facilitate its spread through human activities and offer thoughts on existing knowledge gaps and research needs to mitigate their effects on human health.

## The Environmental Antibiotic Resistome and Mobilome

Bacteria live in a chemical world. They sense and probe the presence of their relatives and neighbors, avoid predators or inhibit the growth of competitors, seek food and shun repellents, and cooperate with and antagonize each other through chemistry. As a result, they collectively have developed a remarkable ability to synthesize bioactive molecules to interact and communicate with other microbes, and in parallel they have developed formidable defense systems to protect themselves against the molecules of others. This reality forms the evolutionary backdrop to the environmental antibiotic resistome: the collection of all the genetic elements that contribute to blocking the action of molecules toxic to bacteria.

The resistome is as ancient as bacterial metabolism, most likely >3 billion years. Recent advances in microbial ecology have revealed the extensive presence of antibiotic resistance genes in environmental bacteria from human polluted, agricultural, and pristine soils. We recognize now that bioactive products and resistance mechanisms in nature are stunningly diverse. Because the scale of microbes on the planet is astronomical in magnitude (>10^30^ bacteria [*6*]), the environmental resistome offers a vast reservoir of genes that have the potential to be mobilized into the (relatively) antibiotic drug–sensitive cadre of bacterial human pathogens that are dwarfed in abundance by their nonpathogenic counterparts.

One of the more distinctive characteristics of microbial genomics is the movement of genes vertically through populations by cell division, and horizontally across species and genera. This movement is enabled by the “mobilome,” the genetic elements that enable and contribute to horizontal gene transfer (HGT) (*7*). Furthermore, antimicrobial drugs—themselves, toxins—and other compounds can favor genetic exchange and increase genetic diversity, thus offering grist for the natural selection mill. Three principal mechanisms are involved in HGT: conjugation (direct cell-to-cell transfer), transduction (phage-assisted transfer), and natural transformation (DNA-to-cell transfer). These mechanisms mobilize genetic elements, such as plasmids, genetic islands, and phages (*8*–*10*), that can contain resistance elements. Furthermore, resistance gene cassettes can be collected by integrons that drive tandem genes from a single promoter element offering stunning multidrug resistance phenotypes (*11*). Additionally, resistance genes can be mobilized within the chromosome and to and from plasmids by transposable elements. The net result is astonishing gene flux in bacterial populations that has no comparator in eukaryotic biology.

The mobilome is key to the spread of genes encoding resistance to antimicrobial drugs and heavy metals and for pathogenic traits among bacteria. Because these functions are often co-located on the same mobile elements, selection for 1 phenotype inadvertently selects for its unintended (and often unrecognized) companion. For example, selection for heavy metal or biocide resistance is often accompanied by antimicrobial drug resistance elements (*4*,*12*–*14*); selection for resistance to 1 drug can co-select for 1 of many others (45 genes in 1 notable example) (*15*). The scale of genetic transfer ranges from short gene segments to megabases of DNA depending on the transfer mechanism involved. Thus, even physically distant genes can be co-selected. These facts form a reality that offers cautionary tales for the substitution of 1 drug for another in response to resistance in a clinical or agricultural setting or for the use of metals (or exposure to them) that can co-select for antimicrobial drug resistance. Furthermore, plasmids can encode toxin/antitoxin systems that result in plasmid addiction even in the absence of selection (*16*,*17*). The net result is an exploded mobile metagenome of shared genetic traits that is fluid and readily promulgated through microbial populations. The rapid movement of water, plants, animals, soil, and humans across the planet virtually ensures that such traits and associated organisms, once easily ecologically segregated, can move seamlessly through habitats across the globe (*18*). The result is that no regions are safe or can escape the introduction and movement of antimicrobial drug–resistant organisms and their genes.

## Environmental Hot Spots of HGT

Retrospective and experimental data demonstrate that so-called hot spots of HGT exist naturally in terrestrial and aquatic environments. These hot spots span scales ranging from micrometer to kilometer: in soil particle pores, in the rhizosphere of plants where root exudates increase the metabolic activity of bacterial populations, or in the residuesphere (the area around decaying plants). Hot spots of HGT in the aquatic environment include air–water interfaces, biofilms formed on multiple surfaces (e.g., the epilithon), and flocs. To these naturally existing hot spots of HGT we now can add environments where human activities have resulted in increased selection pressure, providing the opportunity for resistant phenotypes to multiply. Such environments include sewage treatment plants where a wide range of chemicals meet human and environmental bacteria in high numbers, manure lagoons where bacterial densities and antimicrobial drug concentrations can be very high and exposure periods lengthy, aquaculture ponds that are routinely treated with antimicrobial drugs, biofilters used for degrading pesticides, and environments contaminated by discharge from wastewater treatment plants from antimicrobial drug manufacturing (*19*–*21*).

The enormously high microbial densities, nutrient-rich conditions, and elevated concentrations and chemical diversity of compounds (including antimicrobial drugs) foster HGT in these hot spots. Furthermore, these environments offer ideal conditions for mixing of indigenous saprophytic bacteria and their associated environmental resistome, in a cocktail with high numbers of bacteria of human and animal origin that offer an unprecedented opportunity for genetic exchange between environmental bacteria and pathogens. As noted below, HGT processes can be triggered by various different stresses including chemical exposure and contribute to the adaption of bacterial populations to changing environmental conditions. The result is the exchange and spread of antimicrobial drug resistance well beyond the environmental and clinical settings and ultimately into human microbiomes (*22*). This represents an often silent and potentially deadly reservoir of resistant gut bacteria, which under the right circumstances cause disease in humans that now are recalcitrant to available antibiotic therapies.

## Biotic and Abiotic Selection Pressures of the HGT of Resistance Genes

Abundance is one of several factors influencing the risk that bacteria with acquired resistance will eventually find their way to the human microbiome; the more resistant bacteria we have around us, the greater is the risk we may be colonized by them. The ability of drug-resistant bacteria to propagate in environmental microbial communities in turn depends on selection pressures reducing fitness for competitors lacking relevant resistance determinants. Human activities have led to radically altered selection pressures in various environments through, for example, pollution by antimicrobial drugs. Many of these drugs, including fluoroquinolones, sulphonamides, and tetracyclines, are relatively stable molecules that can persist in water, sediment, or soil long after excretion from humans or animals after therapy, thus providing possible selective advantages to resistant bacteria (*23*). A crucial question is what concentrations of antimicrobial drugs are required to promote resistant strains, because this information will be useful in identifying hot spot environments and in providing a benchmark for mitigation attempts to reduce risks. Controlled competition studies indicate that drug concentrations well below the MICs (i.e., nanograms per liter range) may be sufficient (*24*), but whether this might be translated to more complex microbial communities is not yet clear.

Antimicrobial drug selection is not the only contributor to resistance; exposure of bacterial communities to other chemicals has been conclusively shown to be important in this regard. Indeed, the European Union Scientific Committee for New and Emerging Health Risks concluded that exposure of bacteria to biocides and metals may boost the spread of antimicrobial resistance (*25*,*26*). This interaction is explained by the co-location of resistance genes for antimicrobial drugs and for other chemicals located on mobile genetic elements (as noted above) or by common resistance mechanisms (such as efflux pumps) with a broader specificity (cross-resistance). Biocides with such a potential to promote resistance include chemicals used in health care as disinfectants, antiseptics, or sterilants and on the farm to disinfect equipment (e.g., dairy) and housing (e.g., sheds), as well as preservatives for food and antibacterial agents in household products. Well-established examples are quaternary ammonium compounds, such as the benzalkonium chlorides that form the basis of many commercial and household disinfectants that select for resistance encoded by the *qac* genes, which are frequently recovered on class 1 integrons (*4*) together with antimicrobial resistance gene cassettes. Furthermore, many antimicrobial drugs, metals, and biocides occur together in mixtures in the environment, frequently in hot spots of HGT. The result is a cocktail of selective pressures and biologic responses responsible for enriching resistance in the environment and promoting its transfer to pathogens.

## Dissemination Routes of Resistance Genes and Organisms

Bacteria that acquire resistance genes can be disseminated by multiple routes, and they or their resistance genes ultimately can be acquired by humans and animals. In each situation, multiple clinical and ecologic factors can contribute. Drug-resistant bacteria in humans are eliminated with waste and enter municipal water treatment facilities. From this source, resistance determinants can enter surface waters and affect downstream water supplies through irrigation, runoff, and leaching to the groundwater. Biosolids from sewage treatment facilities, when spread on land, can introduce genes encoding resistance in terrestrial ecosystems. As noted above, these environments are hot spots for HGT, therefore offering great opportunity for genetic exchange between benign environmental saprophytic bacteria and pathogens.

We now have ample evidence for the exchange of antimicrobial drug resistance elements from environmental bacteria and pathogens. Additionally, many human pathogens occur as part of the environment. For example, the presence of enteric bacteria in the water supply, the result of contamination of water with feces, is globally recognized as a fundamental public health concern. Increasingly, these organisms are antibiotic resistant and thus increasingly threaten human health.

## Knowledge Gaps and Research Directions

There is now no debate that the environmental resistome represents a deep reservoir of antimicrobial drug resistance elements that can be readily mobilized into human pathogens resulting in disease that is increasingly challenging to treat. Furthermore, human activities promote favorable conditions for transfer of resistance genes through bacterial populations at previously unprecedented rates. We are approaching a tipping point where multidrug resistance may be too prevalent to halt, let alone reverse. Nevertheless, several important questions remain that need additional research to offer evidence-based responses to this crisis.

1**.** What are the environmental concentrations of antimicrobial drugs, metals, biocides, and other pollutants that are particularly bioactive in the promotion of resistance? What are the concentrations of the compounds that actively amplify resistance and promote HGT, and what are the minimum selective concentrations that must be avoided to decrease the selection for resistance dissemination? The impacts of these agents on microbial physiology and metabolism are largely unknown at concentrations frequently encountered in contaminated environments and most likely are organism and environment dependent.
  1. Are particular mixtures of antimicrobial drugs, metals, and pollutants particularly concerning? Given that microbes are generally exposed to cocktails of anthropogenic compounds, what effects do chemical interactions have on bacterial genetic responses? Evidence is increasing that even combinations of chemical agents can have highly complex and unanticipated effects (both positive and negative) on microbial physiology (e.g., *27*). The genetic co-localization of various resistance and tolerance genes strongly suggests complex interactions between chemicals that cannot be explained by simple concentration, addition or independent action concepts frequently used in predictive mixture toxicology.
  2. What are the environments and micro environments that particularly promote HGT? Can we predict conditions that promote HGT of resistance elements? There is a role here for the prediction of HGT events and their subsequent amplification in the environment by using mathematical models. Development of these models requires decisions on spatial and temporal length scales, which organisms and environmental factors will be considered and which will be ignored, and estimates of contact and transfer rates. The outcomes of such an analysis will begin to identify the critical components that drive resistance dissemination enabling a prioritization of the elements in such systems can be changed (or not) to lessen the impact and slow the spread of resistance.
  3. What is the depth and diversity in the environmental resistome and mobilome? Are particular antimicrobial drugs or classes less susceptible to resistance and HGT? How many resistance genes exist? When new antimicrobial drugs are brought to market should an environmental assessment be conducted to identify the preexisting resistance reservoir? Full understanding of the resistance and HGT genetic landscape should prolong the lifetimes of antimicrobial drugs by identifying resistance before it becomes clinically relevant. This line of investigation also should inform new drug design and development to reduce the opportunity for resistance to emerge.

## Conclusions

We now know that a large, yet largely unclassified, diversity of resistance mechanisms resides within the environmental resistome. Bacteria easily acquire resistance genes, even in the absence of selection, and selective forces can lead to dramatic increases in the prevalence of resistance phenotypes within a microbial community. Hospitals are well known hot spots for the acquisition, amplification, and dissemination of resistance genes because of the steady supply of strong selective pressure through the prevalent use of antibiotic therapy. However, much wider ranges of environments in which genetic exchange of resistance genes and rampant selective pressures are coming together to blunt the effectiveness of the antimicrobial drugs on which we depend. For example, the stacking of genes for resistance to pollutants and multiple antimicrobial drugs on a single mobile genetic element leads to situations in which an array of environmental pressures, largely of human origin, can simultaneously select for many different resistance mechanisms. This is the history of the past 70 years of antimicrobial drug use. The result is a dramatic erosion of our arsenal that is not being replenished. We must act to preserve our current and future ability to control bacterial infection.
